# Efficient and versatile manipulation of the peripheral CD4^+^ T-cell compartment by antigen targeting to DNGR-1/CLEC9A

**DOI:** 10.1002/eji.201040419

**Published:** 2010-03-23

**Authors:** Olivier P Joffre, David Sancho, Santiago Zelenay, Anna M Keller, Caetano Reis e Sousa

**Affiliations:** Immunobiology Laboratory, Cancer Research UK, London Research Institute, Lincoln's Inn Fields LaboratoriesLondon, UK

**Keywords:** Antigen targeting, CLEC9A, DC NK lectin group receptor-1, Immunity, Tolerance

## Abstract

*DC NK lectin group receptor-1* (DNGR-1, also known as CLEC9A) is a C-type lectin receptor expressed by mouse CD8α^+^ DC and by their putative equivalents in human. DNGR-1 senses necrosis and regulates CD8^+^ T-cell cross-priming to dead-cell-associated antigens. In addition, DNGR-1 is a target for selective *in vivo* delivery of antigens to DC and the induction of CD8^+^ T-cell and Ab responses. In this study, we evaluated whether DNGR-1 targeting can be additionally used to manipulate antigen-specific CD4^+^ T lymphocytes. Injection of small amounts of antigen-coupled anti-DNGR-1 mAb into mice promoted MHC class II antigen presentation selectively by CD8α^+^ DC. In the steady state, this was sufficient to induce proliferation of antigen-specific naïve CD4^+^ T cells and to drive their differentiation into Foxp3^+^ regulatory lymphocytes. Co-administration of adjuvants prevented this induction of tolerance and promoted immunity. Notably, distinct adjuvants allowed qualitative modulation of CD4^+^ T-cell behavior: poly I:C induced a strong IL-12-independent Th1 response, whereas curdlan led to the priming of Th17 cells. Thus, antigen targeting to DNGR-1 is a versatile approach for inducing functionally distinct CD4^+^ T-cell responses. Given the restricted pattern of expression of DNGR-1 across species, this strategy could prove useful for developing immunotherapy protocols in humans.

## Introduction

Regulating the T-cell compartment is the principal function of DC and therefore, manipulation of DC offers great promise for immune intervention 1, 2. Numerous studies have focused on the therapeutic potential of *ex-vivo-*generated DC that are subsequently infused into patients 2. An alternative approach consists of Ab-mediated targeting of antigens to endocytic receptors expressed by DC *in vivo* 3, 4. In mice, this method can elicit powerful cellular and humoral responses, beneficial in models of cancer or infection 5–11. Conversely, it can also lead to antigen-specific tolerance, useful for limiting autoimmune diseases or allograft rejection 5, 8, 12–14. Whether antigen targeting to DC results in tolerance or immunity depends on the nature of the targeting Ab, antigen dose, co-administered adjuvants, immunological readout used to measure response, and importantly, the receptor used for targeting 3, 4. Ideally, the latter should be restricted in expression to DC to allow for focused antigen delivery, and should additionally be capable of mediating endocytosis of bound Ab–antigen conjugates and delivering these to antigen processing pathways. In addition, a versatile receptor for antigen targeting should be “neutral” in that its targeting by antibodies should not result in overwhelming delivery of signals that instruct DC to prime particular types of immune responses. Antigen targeting to such “neutral” receptors can then be combined with defined immunomodulators to favor specific immune outcomes, ranging from immunological tolerance to different kinds of immunity.

DC comprise multiple subsets that may be specialized to perform distinct and, sometimes, opposing functions 15, 16. Thus, another consideration in targeting approaches is whether it might be preferable to direct antigens to a single DC subset or to multiple subtypes. Of the large panel of endocytic surface molecules tested as targeting receptors to date, many are expressed by multiple DC subsets and by other populations of hematopoietic and/or non-hematopoietic cells 3, 4. In search for receptors restricted in expression to specific DC subsets, we identified a novel endocytic C-type lectin receptor that we named *DC NK lectin group receptor-1* (DNGR-1) 9, 17, 18. In mice, DNGR-1 (also known as CLEC9A) is expressed at high level by the CD8α^+^ subset and at low level by plasmacytoid DC (pDC) 9, 17, 18. In our studies, mouse DNGR-1 was not detected on other leukocytes, although others have reported low levels of expression on a subset of B cells 17. Interestingly, DNGR-1 expression is also very restricted to DC in human PBMC as it is detected almost exclusively on lineage-negative BDCA-3^+^ cells 9, 17, 18, a subtype of DC proposed to constitute the functionally equivalent of the mouse CD8α^+^ DC population 19. DNGR-1 binds to an unidentified ligand(s) exposed in necrotic cells and is involved in crosspresentation of dead-cell-associated antigens 20. In line with this role, we found that antigens targeted to mouse DNGR-1 *via* antibodies were efficiently crosspresented by CD8α^+^ DC to CD8^+^ T cells 9, 17. Notably, when given with adjuvants, anti-DNGR-1 antigen conjugates induce effective CTL responses that protect mice from tumor challenge in both prophylactic and therapeutic settings 9.

It has recently been shown by Caminschi *et al.* that antigen targeting to DNGR-1 can additionally promote MHC class II presentation and T-cell-dependent Ab production 17. In contrast to CTL priming 9, the Ab responses seen did not require co-administration of adjuvant, suggesting that DNGR-1 targeting to DC might generate intrinsic signals that favor CD4^+^ but not CD8^+^ T-cell priming 17. In this study, we confirm that antigens targeted to DNGR-1 in the steady state can be presented on MHC class II molecules, and we show that this presentation is restricted to CD8α^+^ DC. However, we find that, in the absence of adjuvant, Ab responses are weak and show that this form of antigen targeting does not inevitably lead to CD4^+^ T-cell priming but, rather, can be used to favor the conversion of antigen-specific naïve CD4^+^ T cells into Foxp3^+^ suppressive cells. In contrast, in the presence of adjuvants, the same targeting approach promotes the development of potent Ab and Th1 or Th17 CD4^+^ T-cell responses. Thus, DNGR-1 acts predominantly as a “neutral” receptor, and antigen targeting to this receptor combined with appropriate immunomodulators can be used to promote a wide range of responses, from dominant tolerance to qualitatively distinct types of immunity.

## Results

### Anti-DNGR-1 mAb selectively and rapidly targets CD8α^+^ and pDC in mice

To mark DNGR-1^+^ cells *in vivo*, mice were injected i.v. with fluorophore-labeled anti-DNGR-1 or isotype-matched control mAb. We then analyzed the labeling of different cell types in secondary lymphoid tissues at time points ranging from 5 to 120 min post injection. In mice injected with anti-DNGR-1 mAb but not with the isotype control mAb, we observed rapid and bright staining of the CD8α^+^CD11c^+^ population (Supporting Information Fig. 1A and C). In agreement with the previously described pattern of expression of DNGR-1 9, 17, we were unable to detect any labeling of the CD11c^−^ compartment or CD4^+^ DC, whereas a fraction of pDC was stained, although with reduced intensity and slower kinetics when compared with CD8α^+^ DC (Supporting Information Fig. 1A, B and 2). Systemic inflammation induced by LPS administration did not change the pattern of targeting by anti-DNGR-1 mAb (Supporting Information Fig. 2). These data confirm that anti-DNGR-1 mAb rapidly and specifically targets CD8α^+^ DC and, to a lower extent, pDC.

### Antigen targeting to DNGR-1 in the steady-state allows MHC class II presentation by CD8α^+^ DC

**Figure 1:**
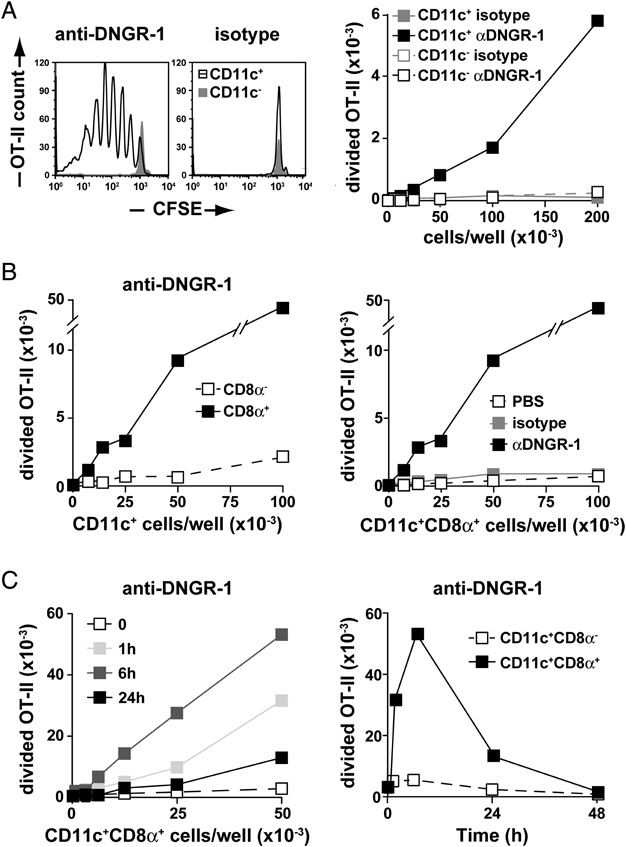
Antigen targeting to DNGR-1 leads to MHC class II presentation by CD8α^+^ DC. (A) B6 mice were injected i.v. with OVA_323–339_-coupled anti-DNGR-1 mAb or with a similarly conjugated Ab control. Four hours later, CD11c^−^ or CD11c^+^ splenocytes were isolated and cultured with CFSE-labeled OT-II cells. OT-II proliferation was assessed by flow cytometry. Representative CFSE profiles are shown in the left panel, whereas the absolute number of divided OT-II cells is represented in the right panel. (B) As in (A) but the CD11c^+^ population was further subdivided into CD8α^−^ and CD8α^+^ fractions. The left panel shows OT-II proliferation induced by CD8α^+^ and CD8α^−^ DC following targeting with anti-DNGR-1 mAb, whereas the right panel shows absolute number of OT-II cells obtained after culture with CD8α^+^ DC isolated from mice injected with PBS, anti-DNGR-1 or control mAb conjugates. (C) B6 mice were challenged with OVA_323–339_-coupled anti-DNGR-1 mAb. At the indicated time points after injection, antigen presentation by CD8α^−^ (right panel) and CD8α^+^ (left and right panel) CD11c^+^ cells was determined as described in (A). For each condition, APC were (A) isolated from two mice and tested separately or (B, C) pooled from two mice and tested in duplicate. Data are the average of duplicate tests and are representative of (A, B) three or (C) two independent experiments.

To test whether DNGR-1 targeting promotes MHC class II antigen presentation by DC, we covalently conjugated anti-DNGR-1 or isotype-matched control mAb to the OVA_323–339_ peptide. We then injected B6 mice with 2 μg of either conjugate and, after 4 h, purified different subpopulations of splenocytes. To reveal processed antigen on MHC class II molecules, we cultured increasing number of cells with CFSE-labeled OVA-specific OT-II CD4^+^ T lymphocytes for 4–5 days. We only observed T-cell division with CD11c^+^ cells purified from mice injected with anti-DNGR-1 mAb (Fig. 1A). Furthermore, among the CD11c^+^ cells, only the CD8α^+^ fraction was able to induce potent OT-II proliferation (Fig. 1B and C). Thus, DNGR-1 targeting allows for MHC class II presentation by CD8α^+^ DC *in vivo*.

MHC class II:peptide complexes generated after targeting to CD8α^+^ DC using DEC205-specific mAb are not stable with time 21. To test whether the same was true when anti-DNGR-1 mAb was used as vector, we injected B6 mice with OVA_323–339_-coupled anti-DNGR1 mAb and analyzed MHC class II presentation by DC at different time points. Consistent with the kinetics of *in vivo* staining, CD8α^+^ but not CD8α^−^ DC were able to efficiently present antigen to OT-II cells as early as 1 h after injection (Fig. 1C). Antigen presentation peaked at 6 h but was markedly reduced by 24 h (Fig. 1C). Thus, antigen targeting to CD8α^+^ DC using anti-DNGR-1 mAb in the absence of adjuvant leads to rapid but short-lived antigen presentation on MHC class II molecules.

**Figure 2:**
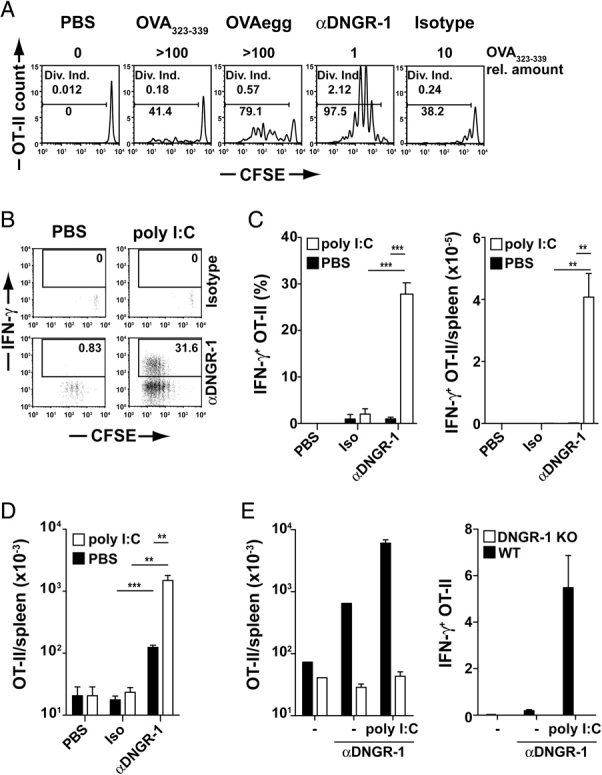
Naïve CD4^+^ T lymphocytes differentiate into Th1 cells following antigen targeting to DNGR-1 in the presence of poly I:C. (A) B6 hosts were infused with CFSE-labeled naïve B6.SJL OT-II cells and injected *i.v.* with either 0.5 μg OVA_323–339_-coupled anti-DNGR-1 mAb (α-DNGR-1), 20 μg OVAegg, 1 μg OVA_323–339_ peptide, 5 μg OVA_323–339_-coupled isotype-matched control or PBS. Five days later, OT-II proliferation was assessed by flow cytometry. Sample acquisition was normalized by time. Bar indicates percentage of divided cells. The division index (Div. Ind.) was determined using the “proliferation” platform of FlowJo. Data are representative of two independent experiments. (B–D) CFSE-labeled naïve B6.SJL OT-II cells were adoptively transferred into B6 hosts. One day later, mice were injected in the footpad with 0.5 μg of OVA_323–339_-coupled anti-DNGR-1 mAb or with a similarly conjugated control (Iso) in the presence/absence of poly I:C. 4–6 days later, OT-II expansion was determined by CFSE dilution and Th1 differentiation was monitored by intracellular IFN-γ staining. (B) Representative flow cytometry profiles are shown. (C, D) Data are the mean±SEM of three independent replicates from a single experiment representative of at least three independent experiments. (E) CFSE-labeled naïve OT-II cells were transferred into either WT or DNGR-1 KO B6 hosts. Mice were then challenged and the CD4^+^ T-cell response analyzed as described in (B). Data are the mean±SEM of three independent replicates from a single experiment representative of two. The *p-*values were determined using unpaired Student's *t*-test (^**^p<0.01; ^***^p<0.001).

To monitor presentation directly *in vivo*, we transferred CFSE-labeled OT-II cells and 1 day later, we injected the mice with 0.5 μg of OVA_323–339_-coupled anti-DNGR-1 mAb, 5 μg of OVA_323–339_-coupled isotype-matched control, 20 μg of OVA (in the form of egg white 22; OVAegg) or 1 μg of OVA_323–339_ peptide. Administering antigen in untargeted form led only to limited proliferation of OT-II cells, while targeting to DNGR-1 resulted in marked cell division and accumulation (Fig. 2A). On a molar basis, we estimate that targeting to DNGR-1 was 10 to 100 times more efficient at inducing CD4^+^ T-cell expansion than delivery of untargeted antigen. Thus, despite the restriction of presentation to a short period of time following antigen delivery (Fig. 1C), DNGR-1 targeting can induce CD4^+^ T-cell proliferation *in vivo*, as recently reported 17.

### Priming of Th1 responses upon antigen targeting to DNGR-1 under the cover of poly I:C

**Figure 3:**
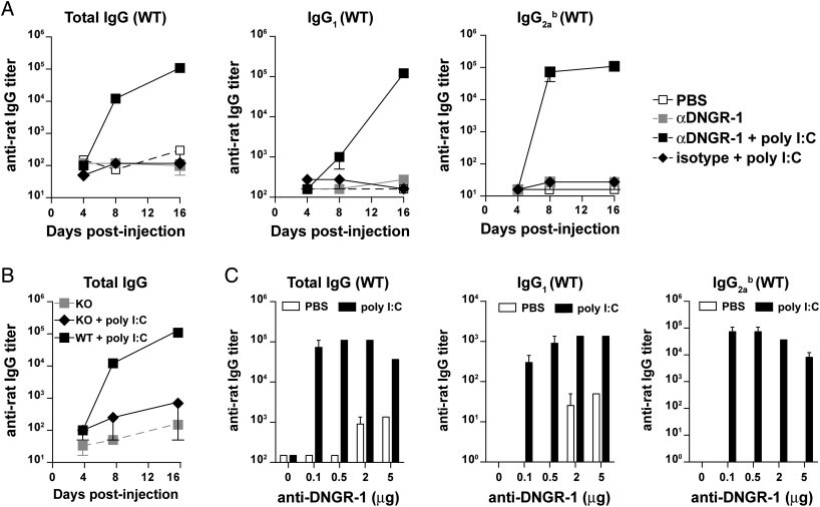
DNGR-1 targeting leads to limited antigen-specific Ab production in the absence of adjuvant WT (A–C) or DNGR-1 KO (B) mice were injected with 0.5 μg (A, B) or with the indicated amounts (C) of anti-DNGR-1 or isotype-matched control mAb in the presence/absence of 40 μg of poly I:C. Anti-rat IgG serum Ab titers were determined (A, B) at the indicated time points or (C) 20 days after immunization. Data are the mean±SD of three mice from one experiment representative of two.

Injection of anti-DNGR-1 mAb did not lead to any detectable activation of splenic CD8α^+^ DC (not shown). Nevertheless, we evaluated whether antigen targeting to DNGR-1 could lead to CD4^+^ T-cell priming in the absence of adjuvant, as recently suggested 17. To avoid any contribution from memory or Treg, we transferred sorted naïve OT-II lymphocytes into B6 mice. One day later, the mice were injected with 0.5 μg of OVA_323–339_-coupled anti-DNGR-1 mAb with or without 40 μg of poly I:C, a TLR3 and RIG-I/MDA5 agonist recently described as the most potent Th1-promoting adjuvant in experiments of antigen targeting to DEC205 23. In the absence of poly I:C, we observed CD4^+^ T-cell expansion but no detectable differentiation into Th1, Th2 or Th17 cells (Fig. 2B and C and data not shown). Consistent with the absence of immunity in these conditions, the mice did not develop a strong Ab response to rat IgG following anti-DNGR-1 injection (Fig. 3A). Low titers of anti-rat antibodies were detected only when injecting a higher dose of anti-DNGR-1 mAb (Fig. 3C), matching the one used in a previous report 17. However, the anti-rat IgG response seen with anti-DNGR-1 alone was dwarfed by that which could be induced by co-administration of poly I:C (Fig. 3C). Poly I:C additionally led to increased expression of MHC class II and co-stimulatory molecules on CD8α^+^ DC (not shown) and strongly boosted the expansion of OT-II cells induced by the Ab–antigen complex (Fig. 2B, D, E). Notably, it also induced robust differentiation of naïve T cells into Th1 effectors, as shown by IFN-γ staining after acute *ex vivo* restimulation with OVA_323–339_ peptide (Fig. 2B, C, E). Demonstrating the specificity of the targeting, no T-cell expansion, Th1 priming or anti-rat IgG response was observed when an isotype-matched control mAb was used (Fig. 2B–D and 3A) or when anti-DNGR-1 conjugates were injected into *clec9a^egfp/egfp^* (“DNGR-1 knockout”; DNGR-1 KO) mice (Fig. 2E and 3B). Th1 differentiation could also be induced with other adjuvants such as anti-CD40 mAb or CpG-containing DNA oligonucleotides (not shown) and could be reproduced in a different adoptive transfer model (Supporting Information Fig. 3). Finally, although CD8α^+^ DC can produce IL-12 in response to innate stimuli, such as poly I:C, identical Th1 responses were seen in WT and IL-12 p40 KO hosts (Supporting Information Fig. 3), confirming that Th1 priming to antigens presented by CD8α^+^ DC is not dependent on IL-12p70 or IL-23 10.

### Differential programming of CD8α^+^ DC by adjuvants leads to differential polarization of CD4^+^ T cells

**Figure 4:**
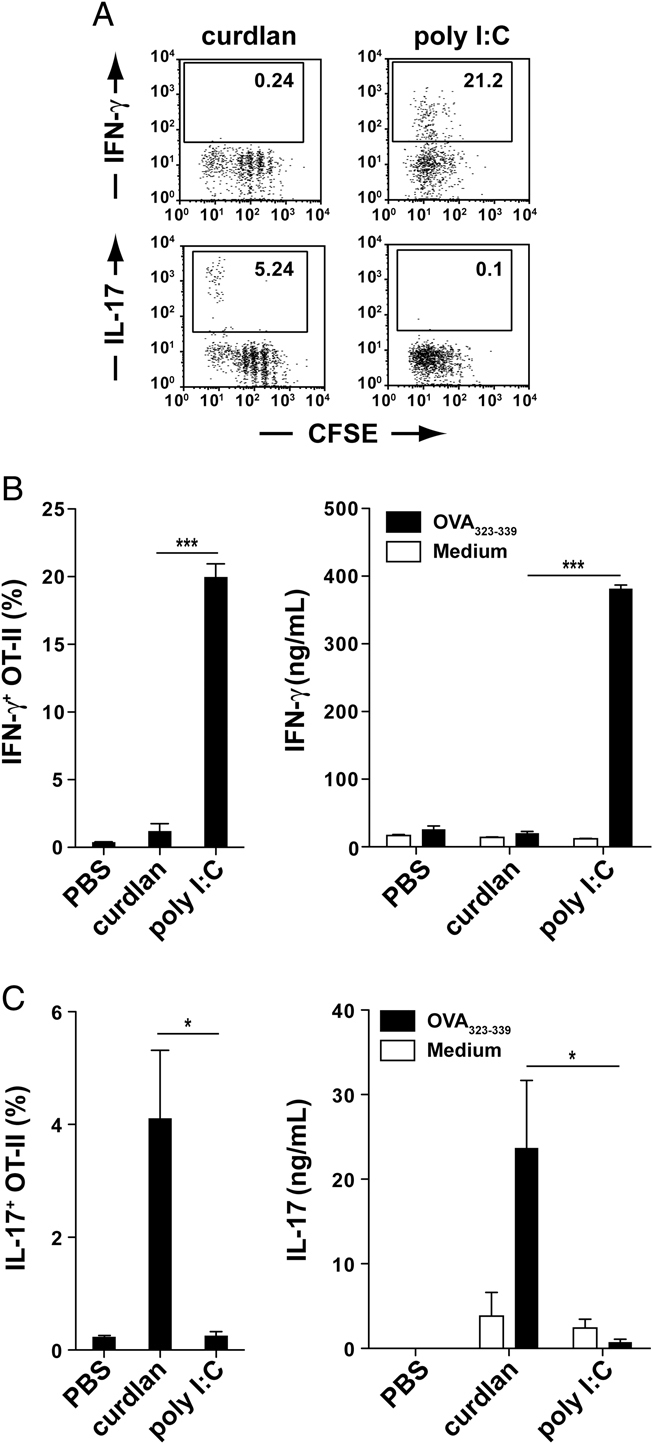
Antigen targeting to DNGR-1 leads to Th17 priming when curdlan is used as adjuvant (A) CFSE-labeled naïve OT-II cells were adoptively transferred into B6.SJL hosts. The day after, mice were immunized with OVA_323–339_-coupled anti-DNGR-1 mAb in the presence/absence of 40 μg poly I:C or 100 μg curdlan. Five days later splenocytes were restimulated with PMA/ionomycin or OVA_323–339_ peptide to measure IL-17 and IFN-γ production by CD45.2^+^ OT-II cells. Analysis was performed by flow cytometry after intracellular staining (A, B and C left panels). (A) Representative stainings are shown. The antigen-specific CD4^+^ T-cell response was also monitored by ELISA after restimulation of splenocytes for 3 days in the presence of OVA_323–339_ peptide (B and C, right panels). (B, C) Data are the mean±SEM of four mice. One representative experiment out of three performed is shown. The *p-*values were determined using unpaired Student's *t*-test (^*^p<0.05, ^***^p<0.001).

DC activated by curdlan, a β-(1, 3)-glucan that acts as a selective Dectin-1 agonist, can steer CD4^+^ T-lymphocyte differentiation into Th17 cells 24. As Dectin-1 is expressed by CD8α^+^ DC 25, we tested whether curdlan could serve as an adjuvant for Th17 priming when antigen was targeted to DNGR-1. B6 hosts received naïve OT-II cells and 1 day later, they were challenged with OVA_323–339_-coupled anti-DNGR-1 mAb together with curdlan or poly I:C. After 5 days, we analyzed OT-II proliferation and differentiation into cytokine-producing cells by flow cytometry and ELISA. Although the use of poly I:C as adjuvant induced a high frequency of IFN-γ^+^ OT-II cells and copious secretion of IFN-γ upon restimulation, curdlan led to minimal differentiation of naïve OT-II cells into Th1 effectors (Fig. 4A and B). Instead, in mice receiving OVA_323–339_-coupled anti-DNGR-1 mAb together with curdlan, OT-II cells differentiated preferentially into IL-17-producing T cells (Fig. 4A and C). These results indicate that DNGR-1 targeting can be harnessed to prime a Th17 response.

### Targeting antigen to DNGR-1 in non-inflammatory conditions can lead to Treg differentiation

**Figure 5:**
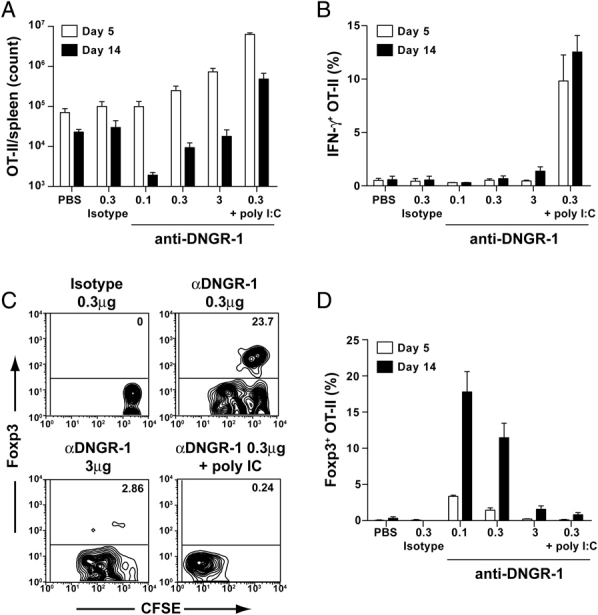
Targeting of small amounts of antigen to DNGR-1 in non-inflammatory conditions leads to the differentiation of Foxp3^+^ cells. CFSE-labeled naïve B6.SJL OT-II cells were adoptively transferred into B6 hosts. The following day, mice were injected with 0.3 μg of OVA_323–339_-coupled isotype-matched control or with indicated amount of OVA_323–339_-coupled anti-DNGR-1 mAb alone or in combination with 40 μg of poly I:C. 5 and 14 days later, (A) the size of the splenic CD45.1^+^ compartment and (B) IFN-γ production after acute restimulation with OVA_323–339_ peptide were determined. (C, D) Foxp3 expression by OT-II cells was also measured by flow cytometry. (C) The flow cytometry profiles are representative from the stainings obtained at day 14. Data are the mean±SEM of 3-9 mice per group from three independent experiments.

In non-inflammatory conditions, antigen presentation by DC can promote differentiation of naïve T cells into Treg 12. To evaluate whether antigen targeting to DNGR-1 could promote Treg conversion, we adoptively transferred naïve OT-II lymphocytes into B6 hosts and 1 day later, injected the mice with different doses of OVA_323–339_-coupled anti-DNGR-1 mAb, alone or in combination with poly I:C. As before, injection of increasing amounts of anti-DNGR-1 mAb led to dose-dependent expansion of the OT-II compartment at day 5 (Fig. 5A) and to significant Th1 differentiation when poly I:C was used as adjuvant (Fig. 5B). Interestingly, a few Foxp3^+^ OT-II cells were detected at this early time point in mice receiving 0.1 or 0.3 μg of anti-DNGR-1 conjugate in the absence of poly I:C (Fig. 5D). The accumulation of Treg became more obvious at 14 days, when 15–20% of the cells expressed Foxp3 (Fig. 5C and D). It was accompanied by a contraction of the OT-II repertoire, greater than the one observed in mice injected only with PBS or with isotype-matched control mAb (Fig. 5A). We conclude that antigen targeting to DNGR-1 in non-inflammatory conditions leads to a strong contraction of the antigen-specific T-cell compartment and allows the peripheral conversion of some remaining naïve T cells into Foxp3^+^ Treg.

## Discussion

Antigen targeting to DC *in vivo* is emerging as an attractive strategy for immunomodulation 3, 4. Ab-mediated delivery of antigenic epitopes to DC has variably been shown to allow priming of CD4^+^ and CD8^+^ T-cell immunity or to induce tolerance through deletion or conversion of antigen-specific T cell into Treg 3, 4. An ideal target should be a surface receptor that delivers the targeting Ab to endocytic and cytosolic compartments for processing of the linked antigenic moiety and subsequent (cross)presentation by MHC class I and/or class II molecules. In addition, it might be desirable to target a “neutral” receptor, *i.e.* one that does not activate DC upon Ab binding, in order to be able to induce tolerance or to tune immunity by co-administering specific immunomodulators. Finally, the target receptor should be restricted to DC, in particular to DC subsets with proved capacity for antigen presentation to T cells. In this study, we show that DNGR-1 fits all of these criteria. DNGR-1-targeted antigens are presented to CD4^+^ T cells selectively by CD8α^+^ DC without promoting strong Th-cell priming. Adjuvants can be co-administered to selectively induce Th1 or Th17 responses. In addition, small amounts of DNGR-1-targeted antigen in the absence of adjuvant can be used to delete antigen-specific T cells and promote Treg conversion.

Although CD8α^+^ DC have been suggested to be less efficient in MHC class II antigen presentation than other DC subtypes 21, this study and many others demonstrate that they are able to present antigens to CD4^+^ T cells *in vivo* 8, 26. They also excel in antigen crosspresentation to CD8^+^ T cells 21, 26, 27 and, therefore, can concomitantly present antigen to both CD4^+^ and CD8^+^ T lymphocytes, allowing optimal delivery of CD4^+^ T-cell help for CTL priming. In addition, as shown here, CD8α^+^ DC can drive the differentiation of Th1 or Th17 cells depending on the adjuvant. Although the ability of CD8α^+^ DC to trigger a Th1 response is well documented, this is the first instance when these cells have been shown to induce Th17 differentiation. These data therefore indicate that CD8α^+^ DC are not ontogenetically pre-programmed to induce Th1 responses and highlight the previously noted importance of innate signals in regulating DC subset function and instruction of adaptive immune responses 28, 29. They further suggest that CD8α^+^ DC are attractive targets for a wide range of vaccination approaches designed to promote immunity to distinct challenges. Interestingly, CD8α^+^ DC can produce large amounts of TGF-β 14. This finding may explain their ability to induce Th17 responses in an inflammatory setting and fits with our previous finding of TGF-β-dependent induction of Th17 cells by curdlan-stimulated DC *in vitro* 25. In addition, TGF-β acts to promote the conversion of naïve T cells into antigen-specific Treg in non-inflammatory conditions 14, 30, 31. This can be seen with small amounts of DNGR-1-targeted antigen in the absence of adjuvant, in agreement with previous conclusions that antigen presentation in sub-immunogenic conditions promotes establishment of tolerance 12. Notably, the CD8α^+^ DC population includes cells able to synthesize retinoic acid, which enhances Treg conversion 32. It is intriguing to speculate that such cells might be responsible for Treg conversion following antigen targeting to DNGR-1. The fact that high doses of antigen and/or strong activation of DC limit Treg accumulation can be explained by the antagonistic effect of T-cell proliferation on the Treg conversion process, as previously reported by Kretschmer *et al*. upon antigen delivery using anti-DEC205 mAb 12.

Tolerance induction by antigen targeting to DNGR-1 could be useful in clinical settings for inducing transplantation tolerance or controlling autoimmunity and could be improved, for example, by co-administering immunomodulatory molecules, such as IL-2 and rapamycin, which expand freshly generated Treg while selectively dampening down the “effector” population 33. It is worth noting that in contrast to the induction of Th1, Th17 or Foxp3^+^ cells, we cannot induce the differentiation of Th2 cells. This result is in line with the notion that CD8α^+^ DC are poor Th2 inducers 34 and fits with recent publications showing that antigen presentation by DC is not involved in driving Th2 responses 35–37. Thus, vaccines or immunotherapies employing antigen targeting to DNGR-1 are unlikely to inadvertently drive a detrimental allergic Th2 response.

We can promote Th1 differentiation with CpG and anti-CD40 mAb but find that poly I:C is by far the most potent inducer of Th1 priming, in agreement with a recent publication 23. Notably, double-stranded RNA, such as poly I:C, triggers IL-12 production in DC 38, but it has been reported that IL-12 is dispensable for Th1 priming when antigen is selectively targeted to CD8α^+^ DC 10. Our finding that anti-DNGR-1 conjugates plus poly I:C prime normal Th1 responses in IL-12 p40-deficient animals is consistent with that report.

Antigen targeting to some DC-expressed C-type lectin receptor has been reported to trigger CD4^+^ T-cell help-dependent B-cell responses in the absence of adjuvant 39, 40. In line with these observations, Caminschi *et al*. recently showed that a single administration of anti-DNGR-1 rat mAb could trigger anti-rat IgG production, and when the mAb was coupled to OVA protein, proliferation of OVA-specific CD4^+^ T cells and an anti-OVA IgG response 17. In their setting, the co-injection of LPS did not boost Ab production and the fact that the humoral response had undergone isotype switching was taken as evidence of CD4^+^ T-cell priming, which was confirmed by using T-cell-deficient mice. When targeting small amounts of antigen to DNGR-1 in the absence of adjuvant, we are unable to induce immunity as assessed by antigen-specific Th1, Th2 or Th17 differentiation or an anti-rat IgG response. Instead, we found that antigen targeting to DNGR-1 in the steady state, if anything, leads to Foxp3^+^ T-cell differentiation. This observation is consistent with the fact that our anti-DNGR-1 antibodies, like those of Caminschi *et al.*, are unable to trigger detectable phenotypic or functional maturation of CD8α^+^ DC, thought to be a prerequisite for immunity 4, 9, 17. With our reagents, inducing an anti-rat IgG response in the absence of adjuvant was only possible when high amounts of antigen were injected. But even when pushing the system in that manner, the response remained 2–3 orders of magnitude lower than the one induced in the presence of poly I:C. These data suggest that antigen targeting to DNGR-1 in the absence of adjuvant might lead to Ab production in certain conditions but that the process is inefficient and that DC activation by a potent adjuvant remains important for triggering of a strong humoral response. Thus, our data largely agree with those of Caminschi *et al.* and any differences might be quantitative and reflect the use of distinct targeting antibodies, possibly bearing different affinities for DNGR-1. The major difference between the two studies is the fact that Caminschi *et al.* found that the inclusion of adjuvant did not substantially boost Ab titers, whereas in our case, we see a massive increase. This discrepancy might be explained by the fact that Caminschi *et al.* used LPS, which is a poor adjuvant in comparison with poly I:C for antigen targeting approaches in which CD8α^+^ DC are the dominant APC (data not shown and 23).

It has recently been proposed that human blood lineage-negative HLA-DR^+^ BDCA-3^+^ cells may encompass functional equivalents of mouse CD8α^+^ DC in mice 19. A genome-wide analysis of the transcriptome of different populations of mouse and human leukocytes supports this contention 41. If BDCA-3^+^ DC prove to have similar properties to the mouse CD8α^+^ DC population, those cells could become attractive targets for immune manipulation. In mice, targeting to DEC205 has been considered as the “canonical” way to direct antigens to CD8α^+^ DC. However, there is no evidence that this lectin is expressed on BDCA-3^+^ DC and additionally human DEC205 has been detected on a large spectrum of hematopoietic cells 3. Given its highly restricted pattern of expression 9, 17, 18, its ability to promote MHC class I crosspresentation and the present demonstration that DNGR-1 targeting can be useful for manipulation of the CD4^+^ T-cell compartment, we envisage that DNGR-1 could constitute a useful target to selectively deliver antigens to BDCA-3^+^ DC and, by extension, for antigen targeting approaches in human.

## Materials and methods

### Mice

C57BL/6 (B6), B6.SJL, OT-II, OT-II B6.SJL and *clec9a^egfp/egfp^* 20 mice were bred at Cancer Research UK in specific pathogen-free conditions. For some experiments, B6 mice were obtained from Charles River. All animal experiments were performed in accordance with national and institutional guidelines for animal care.

### Reagents

Culture medium was RPMI 1640 supplemented with penicillin, streptomycin, HEPES, 2-mercaptoethanol, non-essential amino acids, sodium pyruvate, glutamine (all from Invitrogen) and 10% heat-inactivated FBS (Bioclear). Poly I:C and curdlan were obtained from Amersham and Wako, respectively. OVA_323–339_ peptide was synthesized and purified by HPLC at Cancer Research UK. Sterile-filtered egg white was prepared as previously described 22. The antibodies used for ELISA, specific for mouse IFN-γ (R4-6A2 and XMG1.2 clones) and mouse IL-17 (TC11-18H10 and TC11-8H4.1 clones) were obtained from BD.

### Flow cytometry

Antibodies specific for B220 (RA3-6B2), CD62L (MEL-14), CD25 (PC61), CD44 (IM7), CD4 (RM4-5), CD8α (53-6.7), CD11c (HL3), FcγRIII-II (2.4G2), IFN-γ (XMG1.2), Ly-6G and Ly-6C (RB6-8C5), CD3ɛ (145-2C11) and CD45.2 (104) were obtained from BD. Anti-CD45.1 (A20), anti-Foxp3 (FJK-16s), anti-FR4 (12A5) and anti-IL-17 (TC11-18H10.1) mAb were purchased from eBioscience.

Cell suspensions were blocked with 2.4G2, anti-FCγR washed, resuspended in FACS buffer (PBS, 2 mM EDTA, 2% FBS, 0.2% NaN_3_) containing the appropriate cocktail of antibodies and incubated on ice for 20 min. For intracellular cytokines detection, Fix and Perm® kit (Invitrogen) was used according to manufacturer's instructions. Foxp3 expression was assessed using anti-rat/mouse Foxp3 staining set (eBioscience). Flow cytometry data were acquired on a FACS Calibur or on a LSR II flow cytometer (BD) and were analyzed using FlowJo software (Treestar).

### Generation of Ab–antigen conjugates

Anti-DNGR-1 mAb (7H11, rat IgG_1_) was generated as previously described 9. The Avena phytochrome-specific MAC49 clone was used as isotype-matched control.

Antibodies were activated with sulfo-SMCC (Pierce) and purified by molecular size exclusion chromatography. OVA_323–339_ peptides, with an added cysteine and biotin at the C-terminus (Cancer Research UK), were added and the conjugation reaction was allowed to proceed for 1 h. Conjugates were isolated with GammaBind™ plus Sepharose™ (GE Healthcare). Finally, the number of peptides coupled to each mAb was determined with a Fluoreporter® Biotin Quantitation kit (Invitrogen). The molar ratio between peptides and mAb varied from 1 to 2 but was systematically adjusted between the two antibodies.

### Antigen presentation assay

Mice were injected i.v. with 2 μg of OVA_323–339_-coupled mAb. Four hours later, or at the indicated time points, splenocytes were separated into two fractions using anti-CD11c microbeads (Miltenyi). For further purification, CD11c^+^ cells were labeled with mAb specific for CD11c and CD8α, and the CD11c^+^CD8α^−^ and CD11c^+^CD8α^−^ fractions were sorted on a MoFlo™ cell sorter (Beckman Coulter).

Increasing numbers of APC were co-cultured in round-bottom 96-well plates and in complete medium with 10^5^ CFSE-labeled OT-II cells previously enriched by negative selection using a cocktail of PE-labeled mAb, anti-PE microbeads (Miltenyi) and LD columns (Miltenyi). At day 5, CFSE dilution was determined by flow cytometry. A fixed number of Calibrite™ beads (BD) were added to each sample to quantify the absolute number of OT-II cells *per* well.

### *In vivo* T-cell priming

Naïve CD4^+^ T cells, defined as CD25^−^FR4^−^CD62L^bright^CD44^low^CD4^+^, were purified from CD4-enriched (Dynal® Mouse CD4 negative isolation kit, Invitrogen) spleen and total lymph node cells on a MoFlo™ or FACSAria™ (BD) cell sorter. The population was routinely more than 98% pure and free of Foxp3^+^ cells (not shown). Before transfer, T cells were labeled with 2 μM of CFSE, washed and resuspended in PBS. An amount of 1–2×10^6^ cells were adoptively transferred into CD45.1^+^ or CD45.2^+^ congenic B6 mice. One day later, mice were injected i.v. or in the footpad with indicated amount of OVA_323–339_-coupled mAb alone or in combination with 40 μg poly I:C or 100 μg curdlan. CD4^+^ T-cell responses were assessed 4–6 days after immunization or at the indicated time points. Red-blood-cell-depleted splenocytes were restimulated in complete medium with either 10 μg/mL of OVA_323–339_ peptide or 10 ng/mL of PMA (Sigma) and 1 μg/mL of ionomycin (Calbiochem). After 30 min, Brefeldin A (Sigma) was added to the culture at a final concentration of 10 μg/mL, and the cells were incubated for three more hours. Alternatively, in some experiments, splenocytes were restimulated for 3 days in complete medium with or without 10 μg/mL of OVA_323–339_ peptide. Cytokine accumulation in the supernatant was then monitored by ELISA.

### ELISA detection of anti-rat IgG antibodies

Flat-bottom 96-wells plates (MaxiSorp™ Nunc-immunoplates) were coated with 2 μg/mL of 7H11 mAb. After overnight incubation, unbound mAb was washed away (PBS, 0.05% Tween 20) and non-specific binding sites were blocked with PBS supplemented with 2.5% FBS and 0.2% NaN_3_. Serially diluted sera were then plated and incubated for 6 h at room temperature. After six washes, bound Ab were detected with biotinylated anti-mouse IgG_1_ (B68-2, BD) or anti-mouse 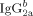 (5.7, BD) mAb or with biotin-SP-conjugated anti-mouse IgG F(ab′)_2_ (Jackson Immunoresearch). Plates were then washed extensively and incubated with extravidin®-conjugated alkaline phosphatase (Sigma). After six washes, the presence of bound Ab was revealed using *p*-Nitrophenyl phosphate (Sigma). Wells were considered as positive when the value of the absorbance measured at 405 nm was superior to the one obtained with the serum from a PBS-injected mouse+3x SEM. The Ab titer corresponds to the last dilution scoring positive.

## Abbreviations

DNGR-1: dendritic cell NK lectin group receptor 1
pDC: plasmacytoid DC
