# Supplementary material for: Efficient and versatile manipulation of the peripheral CD4+ T-cell compartment by antigen targeting to DNGR-1/CLEC9A
Source: Eur J Immunol. 2010 Mar 23;40(5):1255–65. doi: 10.1002/eji.201040419 (PMC3064981; doi:10.1002/eji.201040419)
Supplement: Supplementary file 1 [file eji0040-1255-SD1.pdf]

// European Journal of Immunology

Supporting Information

for

DOI 10.1002/eji.201040419

**Efficient and versatile manipulation of the peripheral $CD4^+$ T-cell compartment by antigen targeting to DNGR-1/CLEC9A**

Olivier P. Joffre, David Sancho, Santiago Zelenay, Anna M. Keller
and Caetano Reis e Sousa

© 2010 WILEY-VCH Verlag GmbH & Co. KGaA, Weinheim 

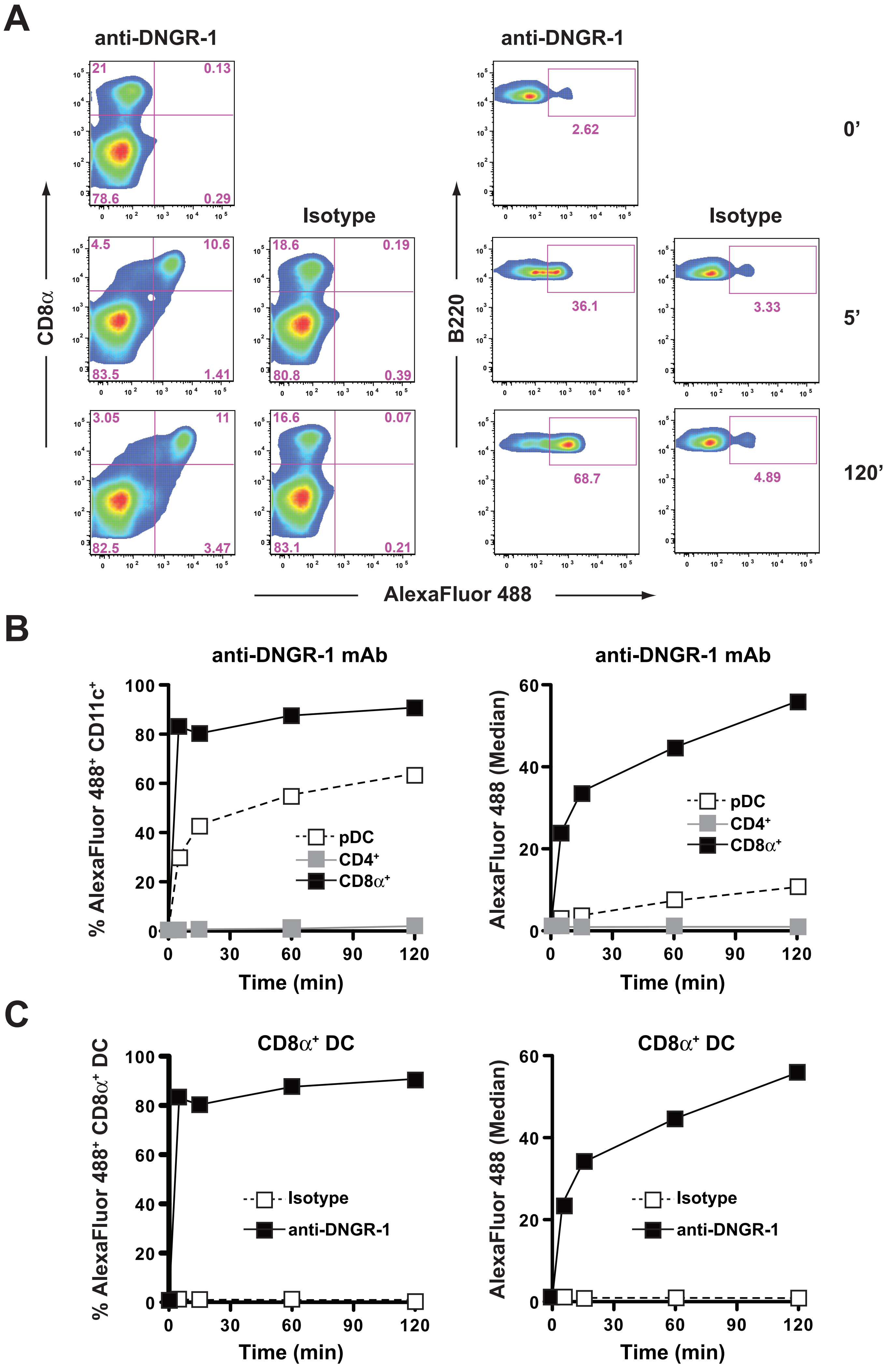


Supplementary Figure 1

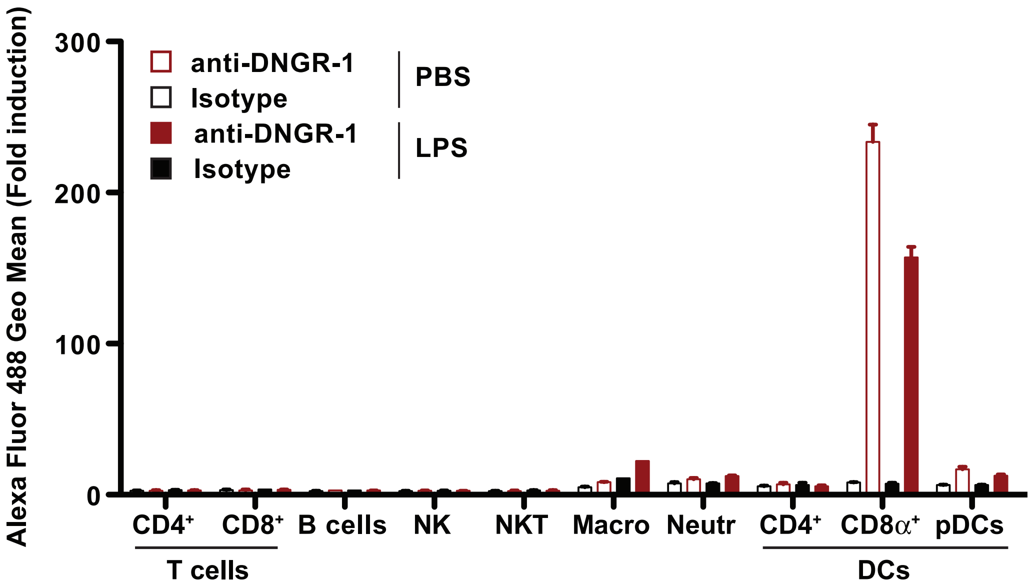


# Supplementary Figure 2

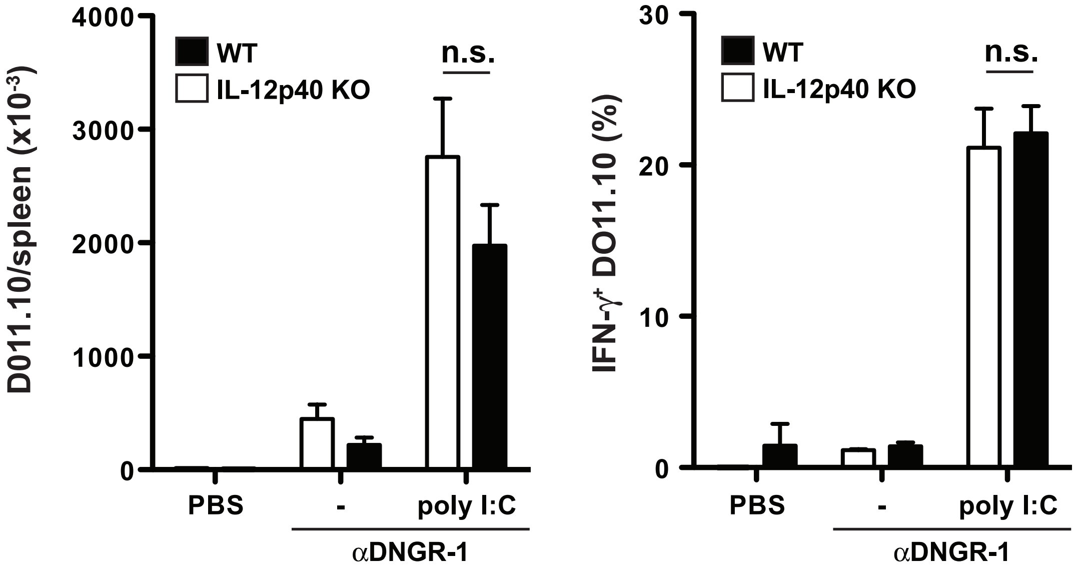


## Supplementary Figure 3

**Supplementary Figure 1 | Anti-DNGR-1 mAb rapidly targets CD8α$^{+}$ DC.**

Anti-DNGR-1 and isotype-matched controls mAb were conjugated to Alexa Fluor 488 using a monoclonal antibody labeling kit (Molecular Probes, Invitrogen). 20μg were then injected *i.v.* into B6 mice. Before injection, or at the indicated timepoints, CD11c$^{+}$ splenocytes were enriched using anti-CD11c microbeads (Miltenyi), stained with the appropriate cocktail of antibodies, and labeling of CD11c$^{high}$CD4$^{+}$ (CD4$^{+}$), CD11c$^{high}$CD8α$^{+}$ (CD8α$^{+}$) and CD11c$^{int}$B220$^{+}$ DC (pDC) was monitored by flow cytometry. Some representative stainings (**A**), the specificity and kinetics of labeling of the different population of DC using anti-DNGR-1 mAb (**B**) and the efficiency of targeting of CD8α$^{+}$ DC by the isotype-matched control or anti-DNGR-1 mAb (**C**) are depicted. In (B) and (C), the left panel shows the percentage of DC labeled by the indicated mAb while the intensity of the staining (median of fluorescence) is represented in the right panel. Data are representative of one out of two independent experiments.

**Supplementary Figure 2 | LPS-induced inflammation does not alter targeting by anti-DNGR-1**

Mice were injected *i.v.* with PBS or LPS (from *Salmonella abortus*, Alexis biochemicals) and 4h later, with Alexa Fluor 488-coupled anti-DNGR-1 or isotype-matched control mAb of irrelevant specificity. 2h later, binding to different subpopulations of spleen cells was analyzed by flow cytometry. Histograms represent the geometric mean obtained with anti-DNGR-1 or isotype-matched control mAb for indicated cell-types.

**Supplementary Figure 3 | Th1 responses induced by DNGR-1 targeting plus poly I:C are IL-12/IL-23-independent.**

Naïve DO11.10 CD4$^{+}$ T lymphocytes purified from DO11.10 SCID BALB/c mice were labeled with CFSE and adoptively transferred into wild-type or IL-12/23 p40 KO BALB/c hosts. 1 day later, mice were injected *i.v.* with 0.5μg of OVA$_{323-339}$-coupled anti-DNGR-1 mAb or isotype-matched control with or without poly I:C. 4 to 6 days later, DO11.10 expansion and differentiation were determined by flow cytometry. Data are the mean ± SEM of all mice (n=6 per group) pooled from two independent.
